# Uric acid as a mediator in the correlation between white blood cells and preeclampsia severity: a retrospective cohort study

**DOI:** 10.1038/s41598-023-47625-4

**Published:** 2023-11-17

**Authors:** Bai-jia Li, Ting-ting Zhu, Xiao-ying Hu, Chao-man He

**Affiliations:** Department of Obstetrics and Gynecology, Sir Run Run Shaw Hospital, Key Laboratory of Reproductive Dysfunction Management of Zhejiang Province, Zhejiang Provincial Clinical Research Center for Obstetrics and Gynecology, Zhejiang University School of Medicine, No. 3 East Qingchun Road, Shangcheng District, Hangzhou, 310018 Zhejiang China

**Keywords:** Hypertension, Risk factors

## Abstract

This study aimed to analyze the independent risk factors for predicting preeclampsia severity and explore its underlying mechanism. Clinical data of patients with preeclampsia were collected from the Medical Information Mart for Intensive Care (MIMIC)-IV database. Univariate and multivariate analyses were employed to assess the significant factors associated with preeclampsia severity. Additionally, we performed multivariate logistic regression analysis and mediation analysis to investigate the potential regulatory path. Based on inclusion and exclusion criteria, 731 participants were enrolled: severe preeclampsia (n = 381) and mild to moderate preeclampsia (n = 350). Age, white blood cells (WBC), platelet, creatinine, albumin, uric acid, aspartate aminotransferase, alanine aminotransferase, international normalized ratio, and prothrombin time were significantly related to preeclampsia severity. Besides, hospital length of stay was significantly higher in the severe group. Notably, age and uric acid were independent predictors for preeclampsia severity. Further, WBC and creatinine were significantly associated with uric acid. Finally, the mediation analysis showed that uric acid was a mediator of the relationship between WBC and preeclampsia severity. In conclusion, WBC might affect preeclampsia severity and progression via the mediation of uric acid. This study might provide novel insight into preventing preeclampsia development.

## Introduction

Preeclampsia is a pregnancy-related disorder defined as new-onset hypertension after 20 weeks of gestation combined with either proteinuria, neurological or hematological abnormalities, liver dysfunction, renal failure, uteroplacental insufficiency, or intrauterine growth restriction^1,2^. Preeclampsia occurs in 5–8% of pregnant women worldwide, which brings a risk of maternal and fetal morbidity and mortality with approximately 60,000 maternal deaths globally^3^. Maternal mortality is even higher when preeclampsia presents with severe manifestations such as visual disturbances and severe headache, hemolysis or eclampsia, decreased platelets, and increased liver enzymes syndrome^4^. Although preeclampsia belongs to one of the common pregnancy complications and several risk factors such as anemia, chronic disease, and parity have been identified as vital predictors for preeclampsia, its exact etiology and pathophysiology remain unclear and may be heterogeneous^5^. Currently, the termination of pregnancy is the only cure for preeclampsia. Nevertheless, mothers with a history of preeclampsia and their children remain at an increased risk of chronic hypertension and future cardiovascular disease^4^. Therefore, it is a serious problem threatening the health of mothers and offspring especially those with severe preeclampsia.

It is generally accepted that preeclampsia is a two-stage model of how inadequate placental perfusion results in a maternal syndrome^6^. In stage 1, decreased placental perfusion leads to aberrant implantation and development of placental vasculature. In stage 2, the women may exhibit an excessive inflammatory response and endothelial cell dysfunction by releasing the placental factors into the maternal circulation^7,8^. Abnormal development of placental vasculature results in placental insufficiency, causing a poor uterine condition and leading to multiple pregnancy complications including preeclampsia^9^. Early diagnosis and close monitoring can help manage preeclampsia during pregnancy to improve maternal and neonatal outcomes^10^. Eclampsia morbidity and associated mortality have been reduced by nearly 90% through early detection during antenatal care and increased access to hospital care for women with preeclampsia^11^. Based on the current findings, we tried to identify the risk factors related to preeclampsia and explore their underlying mechanism. Uric acid was obtained as an independent predictor for preeclampsia in our systematic analysis and white blood cell (WBC) was significantly associated with uric acid. Uric acid presents pro-oxidant and pro-inflammatory properties, and its elevated level may have adverse effects on oxidative metabolism, platelet adhesiveness, hemorheology, and aggregation^12^. Serum uric acid may induce endothelial dysfunction by inhibiting endothelial cell proliferation^13^. However, the mediating role of uric acid in the correlation between WBC and preeclampsia severity was not reported.

This study compared the clinical characteristics and outcomes between severe and mild to moderate groups. Then, the logistic regression analysis was performed to evaluate the independent parameters for preeclampsia severity. Finally, a mediation analysis was conducted to examine whether uric acid could mediate the relationship between WBC and preeclampsia severity.

## Materials and methods

### Data source

This is a retrospective study using the data extracted from the Medical Information Mart for Intensive Care (MIMIC)-IV database, which contains clinical information of patients in the intensive care unit (ICU) at Beth Israel Deaconess Medical Center (BIDMC) from 2008 to 2019. The Institutional Review Boards of BIDMC and the Massachusetts Institute of Technology approved the use of this database. One author, Baijia Li completed the Collaborative Institutional Training Initiative examination (certification number: 56051324) and achieved access to the database. Data extraction was performed using PostgreSQL tools V 12.4.

### Study population

Patients first identified as preeclampsia aged over 18 years were enrolled (n = 1107). The first admission was used for the study analyses if one person had multiple admissions. The exclusion criteria were as follows: discharged within 48 h or died (n = 148); with a history of hypertension (n = 127); with gestational diabetes mellitus (n = 0); with a history of thyroid disease (n = 1); with cardiovascular disease (n = 2); with unspecified preeclampsia (n = 98). Finally, 731 participants were enrolled in the study: severe preeclampsia (n = 381) and mild to moderate preeclampsia (n = 350).

### Data extraction

The following patient characteristics and clinical outcomes were collected: age, body mass index, free T4, thyroid stimulating hormone (TSH), WBC, platelet, creatinine, albumin, total bilirubin, calcium, uric acid, blood urea nitrogen (BUN), aspartate aminotransferase (AST), alanine aminotransferase (ALT), fibrinogen, international normalized ratio (INR), prothrombin time (PT), partial thromboplastin time (PTT), urine volume, potassium, sodium, anion gap, hospital length of stay and ICU stay. Besides, commodities such as hypertension, gestational diabetes mellitus, thyroid disease, and cardiovascular disease were recorded.

### Statistical analysis

Data analyses were performed using the SPSS software (version 23.0) and R (version 4.2.0). Continuous variables were expressed as mean ± standard error or median (quartile) according to the normal distribution and were compared by the Student’s t-test or the Wilcoxon rank-sum test. Categorical data were represented by the numbers (percentages), and the Chi-square or Fisher’s exact tests were used for the comparisons between the two groups. Then, univariate and multivariate logistic regression analyses were adopted to identify that uric acid and age independently predict preeclampsia severity. Following this, patients were divided into two groups based on the median value of uric acid level. The relationship between several clinical characteristics and the uric acid level was analyzed using the Student’s t-test and logistic regression analysis.

Further, the mediation analysis was performed to examine whether uric acid is a potential mediator of the association of WBC with preeclampsia severity. Four steps are required to analyze the mediation effect. First, a significant relation (coefficient: c) between WBC and preeclampsia severity is required. Second, a significant relation (a) between WBC and uric acid is needed. Third, a significant relation (b) between uric acid and preeclampsia severity is required after introducing WBC in the model. Fourth, the coefficient (c) in absolute value relating the WBC to preeclampsia severity should be larger than the coefficient (c’) relating the WBC to preeclampsia severity in the regression model. Fourth, the median effect of the WBC on the preeclampsia severity through the uric acid was evaluated as a × b, and the direct effect of the WBC on the preeclampsia severity was estimated as c’. A P < 0.05 indicated a significant difference.

### Ethical approval and consent to participate

The data we used was extracted from a publicly available critical care database-Medical Information Mart for Intensive Care IV (MIMIC-IV). The privacy of patients in MIMIC IV were protected by using anonymized personal identifier. To protect the privacy of the participants, their identification information was concealed. Therefore, we did not need the specific consent procedures from our institutional ethics committee.

## Results

### General characteristics and clinical outcomes

**Figure 1:**
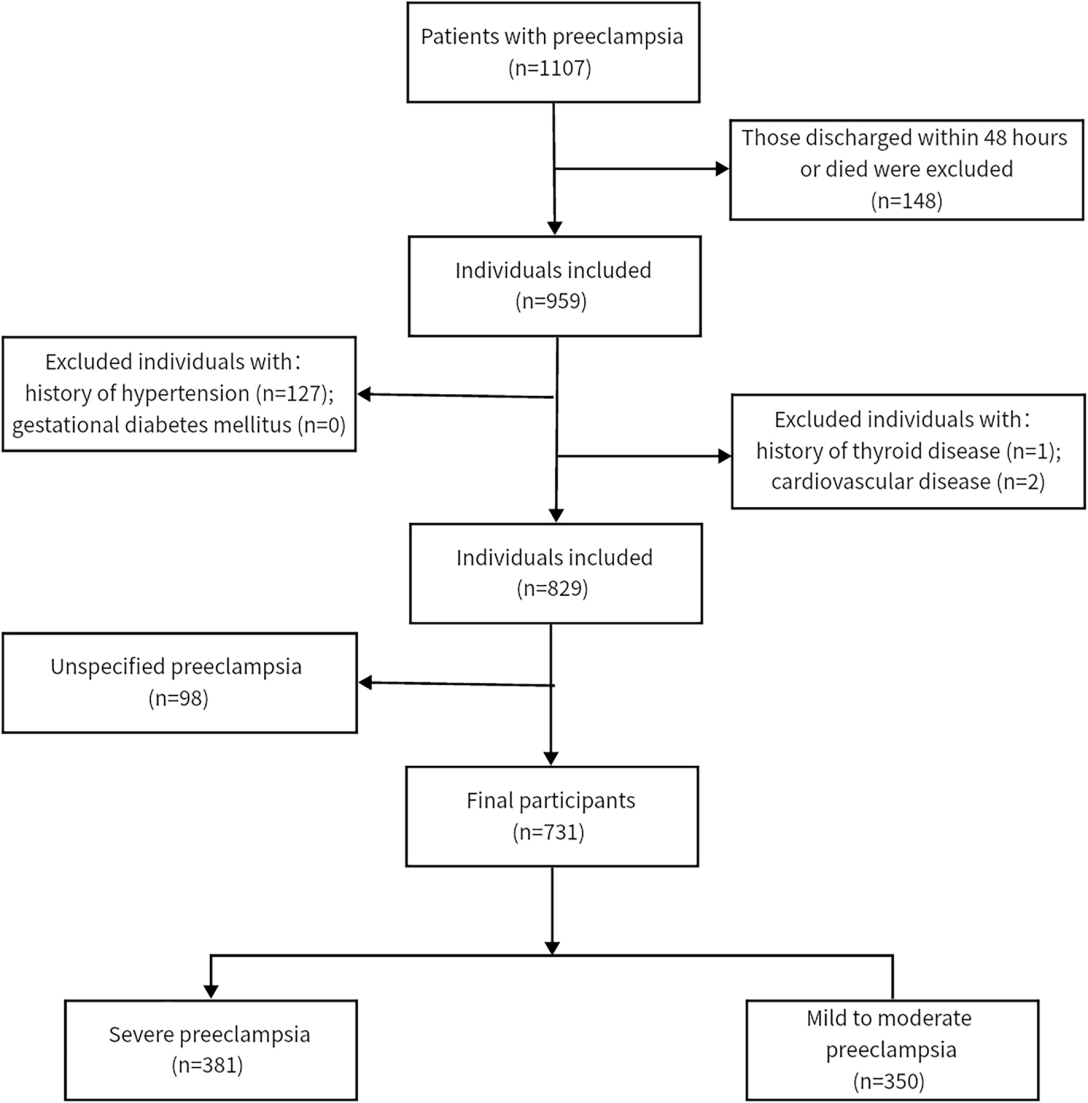
The flowchart of participant selection.

According to the inclusion and exclusion criteria, a total of 731 participants with preeclampsia including severe preeclampsia (n = 381) and mild to moderate preeclampsia (n = 350) were enrolled in the study. Figure 1 illustrates the flowchart of the participant selection and Table 1 summarizes the characteristics of these participants. Those in the severe and mild to moderate groups had an average age of 30.04 ± 0.32 years and 30.99 ± 0.32 years, respectively (P < 0.05). WBC (11.00 ± 0.20 vs 10.38 ± 0.17), creatinine (0.68 ± 0.01 vs 0.64 ± 0.01), uric acid (5.54 ± 0.07 vs 5.33 ± 0.07), AST (49.65 ± 4.16 vs 31.46 ± 2.11), and ALT (45.09 ± 4.63 vs 24.23 ± 2.03) in the severe group were significantly higher than those in the mild to moderate group (all P < 0.05). In addition, platelet, albumin, INR, and PT in the severe group were notably lower compared to the mild to moderate group (all P < 0.05). However, there was no obvious difference in BMI, free T4, TSH, total bilirubin, calcium, BUN, fibrinogen, PTT, urine volume, potassium, sodium, and anion gap between the two groups (all P > 0.05). As was expected, the hospital length of stay was longer in the severe group with statistical significance (P < 0.05). Whereas, no remarkable relationship of ICU stay was observed between the two groups (P > 0.05).

**Table 1:** General characteristics and clinical outcomes of the patients.

Characteristics	Severe preeclampsia (n = 381)	Mild to moderate preeclampsia (n = 350)	P-value
Demographics			
Age (years)	30.04 ± 0.32	30.99 ± 0.32	0.035
BMI (kg/m^2^)	32.89 ± 0.42	32.20 ± 0.44	0.258
Laboratory tests			
Free T4 (ng/dL)	1.31 ± 0.06	1.19 ± 0.03	0.083
TSH (uIU/mL)	3.24 ± 0.99	1.80 ± 0.10	0.148
WBC (K/uL)	11.00 ± 0.20	10.38 ± 0.17	0.017
Platelet (K/uL)	207.18 ± 3.98	218.82 ± 3.39	0.026
Creatinine (mg/dL)	0.68 ± 0.01	0.64 ± 0.01	0.036
Albumin (g/dL)	3.91 ± 0.06	4.10 ± 0.05	0.013
Total bilirubin (mg/dL)	0.46 ± 0.07	0.39 ± 0.03	0.422
Calcium (mmol/L)	8.04 ± 0.15	8.21 ± 0.18	0.471
Uric acid (mg/dL)	5.54 ± 0.07	5.33 ± 0.07	0.030
BUN (mg/dL)	10.76 ± 0.25	11.31 ± 0.30	0.159
AST (IU/L)	49.65 ± 4.16	31.46 ± 2.11	< 0.001
ALT (IU/L)	45.09 ± 4.63	24.23 ± 2.03	< 0.001
Fibrinogen (mg/dL)	472.35 ± 9.29	478.20 ± 10.15	0.673
INR	0.98 ± 0.01	1.00 ± 0.01	0.036
PT (s)	11.00 ± 0.08	11.39 ± 0 .09	0.002
PTT (s)	27.89 ± 0.26	29.41 ± 0.81	0.075
Urine volume (mL)	2405.24 ± 108.98	2476.23 ± 115.88	0.656
Potassium (mEq/L)	4.08 ± 0.03	4.09 ± 0.03	0.919
Sodium (mEq/L)	137.90 ± 0.20	137.98 ± 0.17	0.767
Anion gap (mEq/L)	14.46 ± 0.15	14.64 ± 0.14	0.392
Clinical outcomes			
Hospital length of stay (days)	6.76 ± 0.22	5.94 ± 0.27	0.019
ICU stay (days)	1.97 ± 0.33	2.09 ± 0.53	0.839

*BMI* body mass index, *TSH* thyroid stimulating hormone, *WBC* white blood cell, *BUN* blood urea nitrogen, *AST* aspartate aminotransferase, *ALT* alanine aminotransferase, *INR* international normalized ratio, *PT* prothrombin time, *PTT* partial thromboplastin time, *ICU stay* intensive care unit.

### Uric acid and age independently predict the preeclampsia severity

To identify the independent characteristics for predicting the preeclampsia severity, the identified significant factors including age, WBC, platelet, creatinine, albumin, uric acid, AST, ALT, INR, and PT by the above univariate analysis were enrolled in the logistic regression analysis. In the univariate logistic regression analysis, all the characteristics including age, WBC, platelet, creatinine, albumin, uric acid, AST, ALT, INR, and PT had a close relation with preeclampsia severity (all P < 0.05). In the multivariate logistic regression analysis, age and uric acid could independently predict the severity of preeclampsia (P < 0.05), while other characteristics had no significant association with the disease severity (P > 0.05) (Table 2).

**Table 2:** Factors associated with preeclampsia severity by logistic regression analysis.

Characteristics	Univariate analysis		Multivariate analysis	
	OR (95% CI)	P value	OR (95% CI)	P value
Age (years)	0.975 (0.951–0.998)	0.035	0.942 (0.901–0.985)	0.009
WBC (K/uL)	1.051 (1.008–1.096)	0.019	1.024 (0.957–1.096)	0.496
Platelet (K/uL)	0.998 (0.996–0.999)	0.028	0.998 (0.996–1.004)	0.971
Creatinine (mg/dL)	2.177 (1.036–4.575)	0.040	0.265 (0.053–1.339)	0.108
Albumin (g/dL)	0.672 (0.487–0.927)	0.016	0.705 (0.488–1.018)	0.063
Uric acid (mg/dL)	1.129 (1.011–1.261)	0.031	1.279 (1.002–1.634)	0.049
AST (IU/L)	1.007 (1.003–1.011)	0.001	1.001 (0.989–1.014)	0.846
ALT (IU/L)	1.008 (1.004–1.012)	< 0.001	1.005 (0.995–1.015)	0.353
INR	0.203 (0.045–0.919)	0.038	3.805 (0.033–442.870)	0.582
PT (sec)	0.816 (0.717–0.930)	0.002	0.721 (0.576–1.092)	0.123

*WBC* white blood cell, *AST* aspartate aminotransferase, *ALT* alanine aminotransferase, *INR* international normalized ratio, *PT* prothrombin time.

### WBC and creatinine closely linked to uric acid

Due to the independent role of uric acid and age in preeclampsia severity and the uncontrollability of age, we focused on uric acid and the significant factors associated with preeclampsia severity. The univariate analysis showed that WBC, platelet, creatinine, INR, and PT were significantly related to uric acid (P < 0.05) (Table 3). Further multivariate logistic regression analysis demonstrated that WBC and creatinine were independently correlated with uric acid (Table 4).

**Table 3:** Association of uric acid with the clinical characteristics.

Characteristics	Uric acid (mg/dL)		P-value
	≤ 5.3	> 5.3	
Age (years)	30.35 ± 0.31	30.64 ± 0.33	0.515
WBC (K/uL)	10.39 ± 0.17	11.01 ± 0.20	0.019
Platelet (K/uL)	218.62 ± 3.78	206.96 ± 3.67	0.027
Creatinine (mg/dL)	0.59 ± 0.01	0.73 ± 0.01	< 0.001
Albumin (g/dL)	4.04 ± 0.053	3.96 ± 0.059	0.339
AST (IU/L)	37.95 ± 3.07	44.28 ± 3.79	0.196
ALT (IU/L)	33.16 ± 3.07	37.02 ± 4.27	0.463
INR	1.01 ± 0.01	0.98 ± 0.01	0.009
PT (sec)	11.32 ± 0.09	11.04 ± 0.09	0.027

*WBC* white blood cell, *AST* aspartate aminotransferase, *ALT* alanine aminotransferase, *INR* international normalized ratio, *PT* prothrombin time.

**Table 4:** Association of clinical characteristics with uric acid using multivariate logistic regression analysis.

Characteristics	Odds ratio	95% confidence interval	P-value
White blood cell (K/uL)	1.056	1.002–1.113	0.041
Platelet (K/uL)	0.998	0.996–1.002	0.460
Creatinine (mg/dL)	145.324	38.034–555.264	< 0.001
international normalized ratio	0.173	0.005–6.445	0.342
Prothrombin time (s)	1.021	0.755–1.379	0.894

### Mediation of uric acid in the relation between WBC and preeclampsia

**Figure 2:**
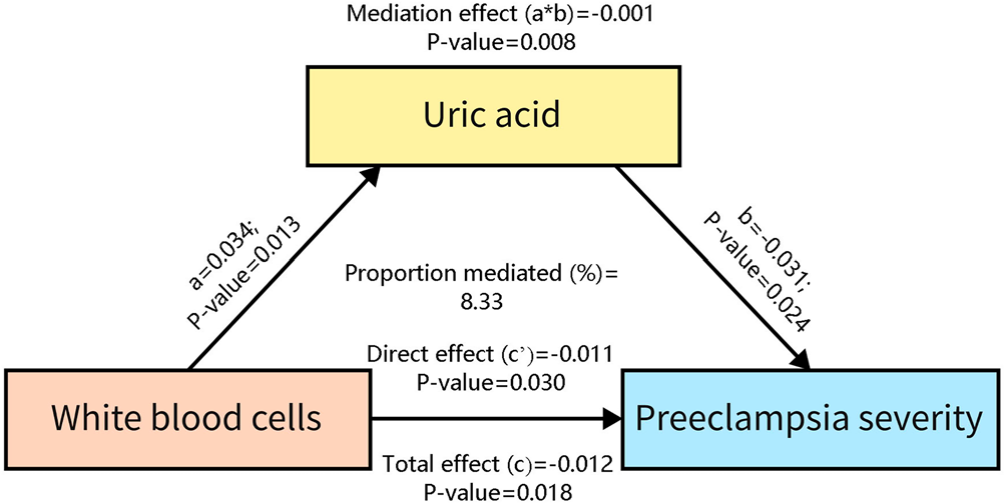
The mediating effect of uric acid on the relation between white blood cells and preeclampsia.

WBC and creatinine were significantly related to uric acid and preeclampsia severity, while uric acid also correlated with preeclampsia severity. When integrating WBC, platelet, creatinine, INR, and PT into multivariate logistic regression analysis, WBC was an independent risk factor of preeclampsia (Table S1). Thus, we performed a mediation analysis to evaluate the mediation effect of uric acid on the association of WBC and creatinine with preeclampsia severity. Uric acid plays a mediating role in the relationship between WBC and preeclampsia severity with a statistical difference (P < 0.05) (Fig. 2). These findings revealed that WBC might affect preeclampsia severity by mediating uric acid.

## Discussion

Herein, we analyzed the significant characteristics associated with preeclampsia severity and revealed that younger age and higher uric acid independently predicted severe preeclampsia. Further univariate and multivariate analyses suggested WBC and creatinine were independent parameters associated with uric acid. Finally, the mediation analysis exhibited that WBC affects the progression of preeclampsia through mediating the uric acid level.

Preeclampsia is a common complication specific to pregnancy and is a major cause of maternal and perinatal morbidity and mortality. Previous studies have demonstrated various environmental and genetic risk factors for preeclampsia severity with diverse results. Therefore, it is valuable and urgent to identify more potential indexes for predicting preeclampsia severity to improve the quality of mothers’ and babies’ lives. Villa et al. showed that earlier preeclampsia in a previous pregnancy, gestational diabetes, obesity, and chronic hypertension increased the risk of preeclampsia occurrence and severe preeclampsia severity^14^. Khadem et al. reported that parity, levels of free T3, free T4, and TSH in the normal pregnant group were not significantly different from those in the preeclampsia group^15^. However, another study showed that notably higher TSH levels were observed in participants with severe preeclampsia than those in the normal group as well as preeclampsia participants without severe features. The upregulation of TSH had an intimate relationship with markers of endothelial dysfunction^16^. Besides, the VEGF—2549 DD genotype, placental—634GC, and CC genotypes were linked to severe preeclampsia^17^. Hence, the authors speculated that changes in THS levels might affect the placental vascular endothelial growth factor, leading to the severity of preeclampsia. Our results showed that both free T4 and TSH were not significantly associated with preeclampsia severity. In addition, significant differences in age, WBC, platelet, creatinine, albumin, uric acid, AST, ALT, INR, and PT were found between the two groups. The difference might be due to the different ethnicity of the enrolled participants.

Subsequently, the multivariate logistic regression analysis showed that uric acid and age could independently predict preeclampsia severity. We further focused on investigating the essential parameters associated with uric acid to elucidate the underlying mechanism of preeclampsia progression. WBC and creatinine were also remarkably connected to uric acid. The mediation analysis confirmed that WBC affected the preeclampsia severity by mediating the uric acid. Normal pregnant women experience a physiological inflammatory response to maintain the immune balance at the maternal–fetal interface, thereby protecting the fetus from disturbance and the mother from abnormal symptoms^18^. Preeclampsia may occur if the immune balance was broken and vascular endothelial cells were damaged by the excessive inflammatory response^19^. Remodeling of the uterine spiral arteries is ascribed to the uterine invasion by trophoblast cells during early pregnancy. Notably, immune cells including mast cells, uterine natural killer cells, dendritic cells, and macrophages are involved in the production of cytokines and other factors in the placental bed. They represent a critical role in immunoregulation, angiogenesis, trophoblast invasion, and spiral artery remodeling^20–22^. In preeclampsia, the function and number of these immune cells may change, which leads to different expressions of relevant factors. This may contribute to a decrease in angiogenesis, trophoblast invasion, and spiral artery remodeling^23,24^. Abnormal invasion of trophoblast cells or destruction of the remodeling of the uterine spiral artery may develop hypoxia/reperfusion, intensify the production of reactive oxygen species (ROS), and trigger oxidative stress in the placenta, which will alter the uric acid production. Notably, WBC including monocytes, lymphocytes, and granulocytes (basophils, eosinophils, and neutrophils) represent a critical role in host defense during tissue injury, infection, and inflammation^25^. The infiltration of WBC could produce and release ROS such as superoxide radical, and hydrogen peroxide via the NADPH oxidase complex, a process also called respiratory burst, resulting in upregulation of oxidative stress, changing the uric acid production^26,27^. This condition will lead to an increase in pro-inflammatory factors synthesis and inflammatory response, aggravating vascular endothelial dysfunction, which in turn promotes the severity and development of preeclampsia^7,8,28–30^. Therefore, it is reasonable to infer that WBC might affect the preeclampsia severity by promoting oxidative stress and changing the levels of uric acid. Attention should be paid to the level of uric acid during prenatal examination. If the level of uric acid is too high, clinicians can recommend timely intervention through diet regulation and other means to prevent preeclampsia.

For strengths, this study investigated the independent factors for preeclampsia severity and excluded participants with related diseases which might affect the accuracy of our results. Besides, we adopted various efficient statistical analyses to explore the underlying mechanism of preeclampsia severity and revealed the potential regulatory path. Although this regulatory path may help direct the clinical prevention for preeclampsia severity classified by symptoms, the classification based on gestational age should be supplemented or validated in future analysis.

In conclusion, age and uric acid were independent characteristics for predicting preeclampsia severity, while WBC and creatinine could independently uric acid. Moreover, WBC might influence preeclampsia severity by mediating the uric acid to induce vascular endothelial dysfunction.

### Supplementary Information

Supplementary Table 1.

## Data Availability

This study analyzed publicly accessible datasets. This data can be found here: https://mimic.mit.edu/docs/.
