# Supplementary material for: Uric acid as a mediator in the correlation between white blood cells and preeclampsia severity: a retrospective cohort study
Source: Sci Rep. 2023 Nov 17;13:20161. doi: 10.1038/s41598-023-47625-4 (PMC10656492; doi:10.1038/s41598-023-47625-4)
Supplement: Supplementary file 1 — Supplementary Table 1. [file 41598_2023_47625_MOESM1_ESM.docx]

| **Table S1** Association of clinical characteristics with preeclampsia using multivariate logistic regression analysis | | | |
| --- | --- | --- | --- |
| Characteristics | Odds ratio | 95% confidence interval | P-value |
| White blood cell (K/uL) | 1.054 | 1.004-1.107 | 0.036 |
| Platelet (K/uL) | 0.997 | 0.994-0.999 | 0.010 |
| Creatinine (mg/dL) | 1.106 | 0.512-2.391 | 0.798 |
| international normalized ratio | 15.074 | 0.514-442.000 | 0.116 |
| Prothrombin time (sec) | 0.669 | 0.504-0.889 | 0.005 |
